# Exercise Training in Aging and Diseases

**Published:** 2012-04-30

**Authors:** Valeria Conti, Giusy Russomanno, Graziamaria Corbi, Amelia Filippelli

**Affiliations:** 1 School of Medicine of University of Salerno, Baronissi (SA), Italy.; 2 Dept of Health Sciences, Faculty of Medicine, University of Molise, Campobasso, Italy.

## Abstract

Sedentary lifestyle along with high blood pressure, abnormal values for blood lipids, smoking, and obesity are recognized risk factors for cardiovascular diseases and for many other chronic diseases, such as diabetes, osteoporosis, breast and colon cancer. Several studies conducted on large cohort of individuals have documented the protective effects of physical activity for both vascular and nonvascular syndromes.

Exercise training is an integral part of cardiac rehabilitation, a complex therapeutic approach, effective both in young and elderly patients. Despite the number of evidences underling the benefits associated with physical fitness, the cardiac rehabilitation is still an underused medical resource.

The molecular mechanism behind physical activity protective effect is presently unresolved, and further studies are also needed to establish the best protocol in terms of specificity, volume and duration of the training.

## INTRODUCTION

Sedentary lifestyle is one of the major risk factors for cardiovascular diseases (CVD) and, for this reason, over last years it has become increasingly important to promote healthy behaviour, including regular physical activity practice^1^.

Exercise Training (ET) has been proven very effective for both prevention and management of many syndromes. It is associated with a reduced risk of type II diabetes development and it is successfully used for secondary prevention of this pathology. One cohort study showed that inactive diabetic patients have a risk of premature death significantly higher than those who practice physical activity 2,3.

Similar evidences were observed in patients with metabolic syndrome and recent studies have demonstrated that more active or fit individuals have a lower amount of total and abdominal fat for a given Body Mass Index (BMI) than their sedentary counterparts 4,5. Thus, the training may play an important role in reducing risk of morbidity and mortality associated with metabolic syndrome, which in turn strictly depends on excess body weight 6.

Other chronic diseases in which the ET was effective are tumors, especially breast and colon cancer, and osteoporosis 7,8. Moreover, it was observed that ET might be very useful to preserve brain structure and function. Indeed, some studies both in animals and in humans suggested that positive environmental factors (cognitive-demanding tasks or physical exercise) favor neuronal plasticity maintenance during aging and protect the nervous system against the eventual damages derived from stress exposure 9.

ET could be an optimal method to preserve aging-related impairment, in particular, central and total obesity, insulin resistance and inactivity increase with age and these factors certainly enhance the risk of all-cause and cardiovascular mortality and of many other chronic diseases. Because ET promote positive changes in body composition and improve insulin sensitivity in older adults, the adoption of a physically active lifestyle should be emphasized in overweight and obese subjects in order to reduce the risk for cardiovascular events in the aging population 10.

At present, the beneficial effects of exercise have been exhaustively demonstrated in CVD prevention and treatment and this is very important because rest and physical inactivity have been recommended to cardiopatic patients for a long time.

### Cardiac rehabilitation (CR)

Physical activity is now considered a valid tool for treatment of cardiovascular diseases so as to justify a real therapeutic approach, named Cardiac Rehabilitation (CR). The CR is a multifactorial process that reduces disability and supports maintenance and recovery of a social active role for patients 11.

ET is an integral part of CR and generally is effective in individuals of both sexes, in young and elderly, in patients with ischemic heart disease either with normal or depressed ventricular function, with clinically stable chronic heart failure and after heart transplantation.

The CR protocols include an exercise program to be continuously carried out, under a close supervision from a medical staff and with a continuous psychological patient’s support 12.

Many authors demonstrated that exercise-based CR guarantees clinical benefits, for instance, physical training improves functional capacity, particularly in individuals with reduced exercise tolerance, and induces an increase in work capacity, maximum oxygen consumption and decrease in heart rate values 13. A long-term exercise-based cardiac rehabilitation leads to changes in myocardial perfusion and function with the absence of unfavorable left ventricular remodeling 14.

Moreover, randomized, controlled exercise trials confirmed the benefit of exercise-based therapy on endothelial function, inflammatory markers, sympathetic neural activation, and metabolism and structure of skeletal muscle 15.

Many evidences clearly shows that patients who participate in CR have several health benefits, including post-hospital care cohordination^16^; improved control of CV risk factors 17,18; reduction of depression and anxiety 19.^21^; and of rehospitalization rates 22,23.

In addition, meta-analyses of randomized controlled trials of CR have demonstrated 15% to 28% reductions in all-cause mortality 24–27. However, these trials included very few elders and cannot represent a frail population. In fact older cardiac patients are often excluded from CR programmes, whereas, benefits of CR and exercise training in functional capacity, in modification of CV risk factors, smoking cessation, antihypertensive therapy and lipid lowering medication has been documented also in older patients, even in those with severe clinical status and multiple co-morbidity conditions 28.

In conclusion, despite a large amount of data collected in support of its efficacy and the guidelines from American Heart Association (AHA) and American College of Cardiology (ACC) for secondary prevention of coronary heart disease and other vascular syndromes, CR remains an underutilized medical resource 29. The reasons are cultural, organizational and economic and have been extensively analyzed 30. Doubtless, one of the most important problems is the lack of adequate information about the CR real benefits that should be addressed both to medical staff and patients.

### Exercise training molecular effects

Up to now, very little it is known about molecular pathways in which exercise training is implicated.

ET certainly can induce an accumulation of oxygen reactive species (ROS), which often leads to oxidative stress. However, it is very important to distinguish between acute and chronic exercise and it is essential to consider together the type (aerobic or anaerobic), the intensity and duration of physical activity.

Over the last thirty years it has become increasingly evident that the benefits of physical exercise in humans, but also in animals used as an experimental model, are due to its ability to induce favorable cellular adaptations. In fact, it is now believed that the ROS production could be a “signal” necessary to stimulate the body's physiological antioxidant systems. In other words, while the signal of a stimulus is given in acute, beneficial cellular adaptations are permitted and supported only by a chronic exercise 31.

Ferrara et al demonstrated that moderate and prolonged exercise training favored antioxidant system and induced the activity of Sirtuin 1 (Sirt1), an enzyme possessing either histone deacetylase or mono-ribosyltransferase activity, in the heart and adipose tissue of aged rats. The authors concluded that ET, by antioxidant endogenous system and Sirt1 activity induction, could counteract age-related systems impairment 32.

To date, it has not been unequivocally demonstrated that excessive production of oxidants, due to both aerobic and anaerobic activity of high intensity, guide necessarily to a biological damage 33,34. On the contrary, moderate exercise has proved useful to trigger different physiological adaptations. In summary, during the training sessions, repeated exposure to stressful stimuli resulting from the ROS accumulation would lead to overwhelm the antioxidant defense with a shift of the redox balance to a reducing cellular environment, which in turn favors the defense mechanisms.

These evidences are consistent with the concept of “hormesis”. The term of hormesis (etymologically, stimulation) in biology and medicine indicates an adaptive response of cells and organisms to a moderate and, usually, intermittent stress.

**Figure 1:**
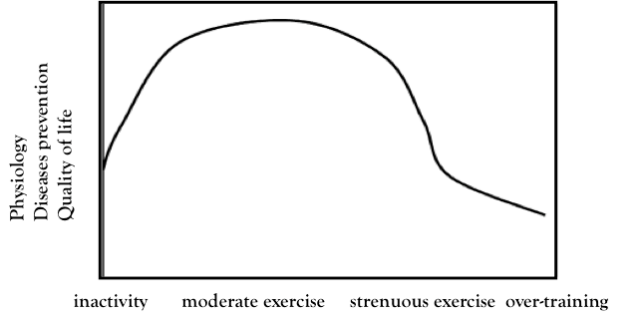
U-curve describes hormetic effects of exercise training.

Regular physical activity is properly an inducer of intermittent stressful stimuli and the response of biological systems to ET can be described with a U-curve, which relates generic indicators of quality of life with a range of physical activity that goes from inactivity to over-training 35,36 (Figure 1).

One of the most important adaptive responses associated with the ET regards the mitochondrial energy metabolism stimulation. This event involves the transcriptional regulation of genes such as peroxisome proliferator-activated receptor gamma (PPAR-gamma), the peroxisome proliferator-activated receptor-Coactivator (PGC-1alpha) and the transcription factor A mitochondrial (TFAM) 37.

Recently it was shown that many molecules are involved in the hormetic response. They are ion channels, kinases and deacetylases and transcriptional factors that regulate the expression of genes encoding cytoprotective proteins. Among these proteins, molecular chaperones (heat shock proteins, Hsps), antioxidants such as superoxide dismutase and glutathione peroxidase, and growth factors such as insulin factor 1 (IGF1) and neurotrophic factor (BNF) have been identified 38–40.

It is very important to underline that exercise-induced hormesis may help healthy aging. Indeed, aging is a complex physiological process associated with a significant decrease in physical activity attitude with loss of muscle mass and ability to movement. It has been shown that regular exercise can help to reach what is referred as “successful aging” delaying and reducing the inevitable manifestations of functional decline 41.

Many studies have shown that physical inactivity increases the incidence of age-associated diseases^42^ and that exercise promotes beneficial effects in several organs by reducing the level of ROS, postponing aging and decreasing the incidence of diseases associated with it^43^,^31^.

Although significant progresses, many difficulties remain towards understanding the mechanisms underlying the effects of exercise in humans. The limitations mainly depend on the fact that the vast majority of research in the field have used aerobic protocols with sub-maximal or maximal effort and based on short or moderate duration^32^. In addition, there are very few studies that have compared different types of exercise, and almost all are based on the comparison between the effects of a type of exercise and a “control” represented by a group of sedentary individuals.

Until now, very little has been demonstrated on the molecular pathways associated with the antioxidant effects of exercise especially in regard to the type and volume of exercise.

### Exercise training specificity

**Figure 2:**
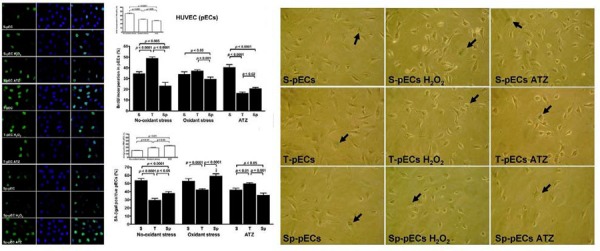
Proliferation rate by BrdU staining (panel on the left) and senescence levels by ß-Gal assay (panel on the right) in primary endothelial cells (HUVEC) conditioned with sera from athletes practicing aerobic (thriatlon, T), mixed (soccer, S) and anaerobic (sprint run, Sp) exercise trainings, with or without H_2_O_2_ stress induction (*Conti V. et al, Med Sci Sports Exerc. 2012;44:39–49, with permission)*.

Recently, Conti et al. compared the amount and quality of response to oxidant/antioxidant activity in sera of athletes practicing continuously (at least five years) different sports, i.e. aerobic, mixed and anaerobic exercise trainings and used an in vivo-in vitro technique to evaluate the effects of sera isolated from athletes in the human endothelial cells (ECs) 44. In the ECs conditioned with athletes’ sera and submitted or not to induction of oxidative stress (with hydrogen peroxide) some indicators of cell viability, as survival, proliferation and senescence and the expression and activity of markers of oxidative stress were evaluated. The authors indicated the aerobic exercise as the best exercise protocol able to promote the release of circulating factors which, in turn, induce favorable adaptations and an organism's response to stressful stimuli (Figure 2).

Application of the in vitro–in vivo technique (i.e., cultured cells incubated with fresh human serum) used in that study provided probably for the first time direct evidence that human serum strongly affects redox homeostasis of endothelial cells and that these effects are dependent on the exercise training stimulus.

Moreover, the study shifts the attention from the classic muscle-centric to the blood-centric vision on the control of the human body redox state 45. It is very important to stress that available literature data remain still controversial especially in regard to the intensity of exercise required to achieve significant protective effects. For instance, Farsidfar et al. reported that an intense training induces an immediate change in endothelial function. Other authors have shown that strong exercise, as related to a very high oxygen consumption, favors the ROS accumulation and generates oxidative stress, particularly at the skeletal and cardiac muscles 46,47. However, also in this case overall data do not allow to reach definitive conclusions, could be very useful to perform new studies to clarify this issue.
